# Metabolic profiling and targeted lipidomics reveals a disturbed lipid profile in mothers and fetuses with intrauterine growth restriction

**DOI:** 10.1038/s41598-018-31832-5

**Published:** 2018-09-11

**Authors:** Jezid Miranda, Rui V. Simões, Cristina Paules, Daniel Cañueto, Miguel A. Pardo-Cea, María L. García-Martín, Francesca Crovetto, Rocio Fuertes-Martin, Monica Domenech, María D. Gómez-Roig, Elisenda Eixarch, Ramon Estruch, Stefan R. Hansson, Nuria Amigó, Nicolau Cañellas, Fatima Crispi, Eduard Gratacós

**Affiliations:** 1 Fetal i+D Fetal Medicine Research, BCNatal – Barcelona Center for Maternal-Fetal and Neonatal Medicine (Hospital Clínic and Hospital Sant Joan de Deu), Institut Clinic de Ginecologia, Obstetricia i Neonatologia, IDIBAPS, University of Barcelona, and Centre for Biomedical Research on Rare Diseases (CIBER-ER), Barcelona, Spain; 2 Metabolomics Platform, IISPV, DEEiA, Universidad Rovira i Virgili, Tarragona, Spain; 3 Biosfer Teslab, Reus, Spain; 4 BIONAND, Andalusian Centre for Nanomedicine and Biotechnology, Junta de Andalucía, Universidad de Málaga, Málaga, Spain; 5 Hospital Clínic, Institut d’Investigacions Biomèdiques August Pi i Sunyer, University of Barcelona, Barcelona, Spain; 6 CIBER Fisiopatología de la Obesidad y Nutrición (CIBEROBN), Instituto de Salud Carlos III, Madrid, Spain; 7 Department of Obstetrics and Gynecology, Institute of Clinical Sciences, Lund University, Sweden; Skåne University Hospital, Lund, Sweden; 8 CIBERDEM, Spanish Biomedical Research Centre in Diabetes and Associated Metabolic Disorders, Madrid, Spain

## Abstract

Fetal growth may be impaired by poor placental function or maternal conditions, each of which can influence the transfer of nutrients and oxygen from the mother to the developing fetus. Large-scale studies of metabolites (metabolomics) are key to understand cellular metabolism and pathophysiology of human conditions. Herein, maternal and cord blood plasma samples were used for NMR-based metabolic fingerprinting and profiling, including analysis of the enrichment of circulating lipid classes and subclasses, as well as the number of sub-fraction particles and their size. Changes in phosphatidylcholines and glycoproteins were prominent in growth-restricted fetuses indicating significant alterations in their abundance and biophysical properties. Lipoprotein profiles showed significantly lower plasma concentrations of cholesterol-intermediate density lipoprotein (IDL), triglycerides-IDL and high-density lipoprotein (HDL) in mothers of growth-restricted fetuses compared to controls (p < 0.05). In contrast, growth-restricted fetuses had significantly higher plasma concentrations of cholesterol and triglycerides transporting lipoproteins [LDL, IDL, and VLDL, (p < 0.005; all)], as well as increased VLDL particle types (large, medium and small). Significant changes in plasma concentrations of formate, histidine, isoleucine and citrate in growth-restricted fetuses were also observed. Comprehensive metabolic profiling reveals that both, mother and fetuses of pregnancies complicated with fetal growth restriction have a substantial disruption in lipid metabolism.

## Introduction

Fetal growth restriction (FGR) affects 6–10% of all pregnancies and is defined as the failure to achieve the genetic growth potential, resulting in a given low birthweight^1^. Growth restricted fetuses have a 5 to 10-fold risk of dying *in utero*, and higher risk of perinatal morbidity and mortality^2,3^. In addition, fetuses with growth restriction show metabolic and cardiovascular adaptations that are thought to persist postnatally, with implications for adult disease and repercussions for preventive strategies. A small fraction of the small fetuses diagnosed in utero present as early-onset/severe fetal growth restriction^4^. However, the majority of clinical instances of fetal smallness occur late in gestation under two main phenotypes, conventionally defined as small for gestational age (SGA) and FGR^5,6^. While the former is usually associated with near-normal perinatal outcomes and are considered “constitutionally small fetuses”, FGR is characterized by placental maldevelopment and abnormal fetoplacental blood flow^7^, a higher risk of fetal death^3,8–10^ and poor perinatal outcomes^11,12^. It is unclear whether SGA and FGR are different conditions or represent different degrees of severity of the same disease. For instance, despite the clear differences in obstetrical risks, both SGA and FGR show similar features of long-term fetal adaptation to intrauterine undernutrition, including signs of cardiac remodeling^13–17^, differences in brain cortical development^18–20^ and microstructure^21–23^, as well as a higher prevalence of neurodevelopmental delay^24–27^.

The metabolome of biological fluids represents a sensitive and multifactorial phenotypic signature of disease, providing insights of the interface between the final downstream products of gene expression, the influence of environmental factors and the complex interactions between them^28–31^. In addition, recent sensitive, rapid, and high-throughput technology advances have provided a solution to measure low concentrated metabolites in human plasma, allowing a comprehensive profiling of metabolic changes *in-vivo*^32–34^. Fetal growth may be impaired by poor placental function or maternal conditions, each of which can influence the transfer of nutrients and oxygen from the mother to the developing fetus^35^. We and others have previously explored the metabolomic profile of fetuses with impaired intrauterine growth^36–39^. Specifically, a metabolomic analysis of cord blood from early and late-onset FGR revealed significant differences in essentials amino acids as well as an abnormal lipid metabolism in both, early and late-onset FGR; particularly at the expense of unsaturated lipids^38^. However, the enrichment of circulating lipid classes and subclasses, as well as the number of sub-fraction particles and their size, has not been analyzed in detail. Furthermore, the co-existence of metabolic changes in the maternal compartment has not been explored. A simultaneous and comprehensive characterization of the metabolomic profile of mothers and fetuses with suboptimal fetal growth could provide further insights into the pathophysiological changes underlying these clinical forms of fetal smallness. The objective of this study was to analyze the maternal and cord blood plasma metabolome in pregnancies with two clinical phenotypes of late-onset fetal smallness, SGA and FGR, and to compare them with those from pregnancies with a normal fetal growth.

## Results

### Clinical characteristics of participants

From October 2014 to March 2016, 80 pregnant women were recruited. Among those, 28 had a normal sonographic estimation of fetal weight that was confirmed at birth [Adequate-for-gestational age fetuses (AGA)] and 52 pregnancies had an antenatal diagnosis of fetal smallness [estimated sonographic fetal weight (EFW) below the 10^th^ centile] that was confirmed at birth (birthweight <10^th^ centile) and were included as cases. According to our clinical protocol, small fetuses were subdivided as follows: those with an EFW <3^rd^ centile (confirmed at birth) and/or abnormal uteroplacental flow defined by an abnormal uterine artery Doppler velocimetry and/or abnormal cerebroplacental ratio (a sonographic Doppler parameter that indicates redistribution of fetal cerebral blood flow) were termed *fetal growth restriction* (FGR; n = 27), while those with a birthweight between the 3^rd^ and the 9^th^ centile and normal fetoplacental Doppler were considered *small-for-gestational age* (SGA; n = 25) cases^5,40,41^.

The maternal sociodemographic and obstetric characteristics are summarized in Table 1. There were no significant differences in maternal age, race, smoking or rate of maternal underweight [pre-gestational body mass index (BMI) <18 kg/m^2^] or obesity (BMI >30 kg/m^2^) among the study groups (all p values > 0.05). Furthermore, there were no significant differences between SGA and FGR (p = 0.07) or AGA (p = 0.21) when we analyze pre-gestational BMI as a continuous variable (Table 1).

**Table 1:** Clinical characteristics of the study groups.

	Adequate for Gestational Age N = 28	Small for Gestational Age N = 25	Fetal Growth Restriction N = 27	p value
	n (%) or median (IQR)	n (%) or median (IQR)	n (%) or median (IQR)	K-wallis
***Maternal baseline characteristics***				
Maternal age (years)	32 (30.5–36.5)	30 (25–35)	31.5 (28–34)	0.16
Maternal race				0.77
White	23 (82.1)	19 (79.2)	24 (92.3)	
Latin	2 (7.1)	3 (12.5)	1 (3.8)	
Indian/Pakistan	2 (7.1)	1 (4.2)	1 (3.8)	
Others	1 (3.6)	1 (4.2)	0	
Pre-gestational maternal BMI (kg/m^2^)				0.21
< 18.5	1 (3.57)	1 (4.3)	5 (20)	
18.5–25	19 (67.9)	17 (74)	16 (64)	
25–30	5 (17.9)	5 (21.7)	4 (16)	
> 30	3 (10.7)	0	0	
Smoking	4 (14.2)	8 (32)	5 (18.5)	0.3
***Ultrasound parameters at third trimester evaluation***				
GA at recruitment (weeks)	32.3 (30–34.8)	33.8 (31.3–36.4)	34.4 (32–36)	0.19
Estimated fetal weight (grams)	2131 (1477–2615)	1866 (1406–2134)	1788 (1567–2040)	0.24
Estimated fetal weight centile	50 (39–77)	5 (3–9)	3 (1–6)	0.0001
Mean uterine artery PI (Z-score)	−0.4 (−1.1–0.67)	0.2 (−0.14–0.78)	1.05 (−0.86–2.02)	0.15
Mean uterine artery PI > 95^th^ centile	1 (4.2)	0	9 (36)	0.001
Umbilical artery PI (Z-score)	−0.26 (−0.58–0.29)	−0.1 (−0.68–0.4)	0.44 (−0.1–0.65)*	0.02
Umbilical artery PI > 95^th^ centile	0	0	3 (11.1)	0.1
Cerebroplacental ratio (Z-score)	−0.16 (−0.7–0.47)	−0.51 (−1.43–0.16)	−1.08 (−1.45–0.11)*^¥^	0.16
Cerebroplacental ratio < p5^th^ centile	3 (12)	0	5 (18.5)	0.74
***Perinatal outcomes***				
GA at delivery (weeks)	39.8 (39–40.5)	39.4 (38.1–40.3)	37.8 (37.3–39.6)*^¥^	0.002
Birthweight (grams)	3365 (3060–3575)	2728^¥^ (2500–2860)^¥^	2245 (2100–2506)*^¥^	0.0001
Birthweight centile	42 (35.5–68.5)	6 (4–9)^¥^	1 (0–2)*^¥^	0.0001
Male gender	11 (39.3)	11 (44)	19 (70.4)	0.05
Induction of labor	6 (21.4)	13 (52)	18 (66.7)	0.003
Route of delivery				0.67
Vaginal delivery	18 (64.3)	11 (44)	15 (55.6)	
Cesarean section	9 (32.1)	12 (48)	10 (37)	
Operative vaginal delivery	1 (3.6)	2 (8)	2 (7.4)	
Preeclampsia	0	0	3 (11.1)	0.9

BMI: Body mass index; GA: Gestational age; PI: Pulsatility index;

Missing values: Race (2); BMI (4); Uterine artery Doppler velocimetry (6); Umbilical artery Doppler velocimetry (4) and cerebroplacental ratio (4).

*Statistically significant different (p < 0.05), between SGA and FGR. ^¥^Statistically significant different as compared to AGA.

According to the design of the study, there were no significant differences in the gestational age at recruitment, cases had a significantly lower estimated sonographic fetal weight compared to controls and all patients delivered at term (>37 weeks of gestation). FGR cases had significantly higher rate of abnormal uterine artery Doppler and worse fetoplacental Doppler parameters as compared to controls and SGA. There were no significant differences in the gestational age at delivery between SGA cases and controls (p = 0.15); however, it was significantly lower in FGR cases (p = 0.002). The rate of induction of labor was significantly higher in cases; nevertheless, there were no differences in the route of delivery or rate of preeclampsia among the study groups (Table 1).

### ^1^*H-NMR* spectral fingerprinting in maternal and cord blood spectra

**Figure 1:**
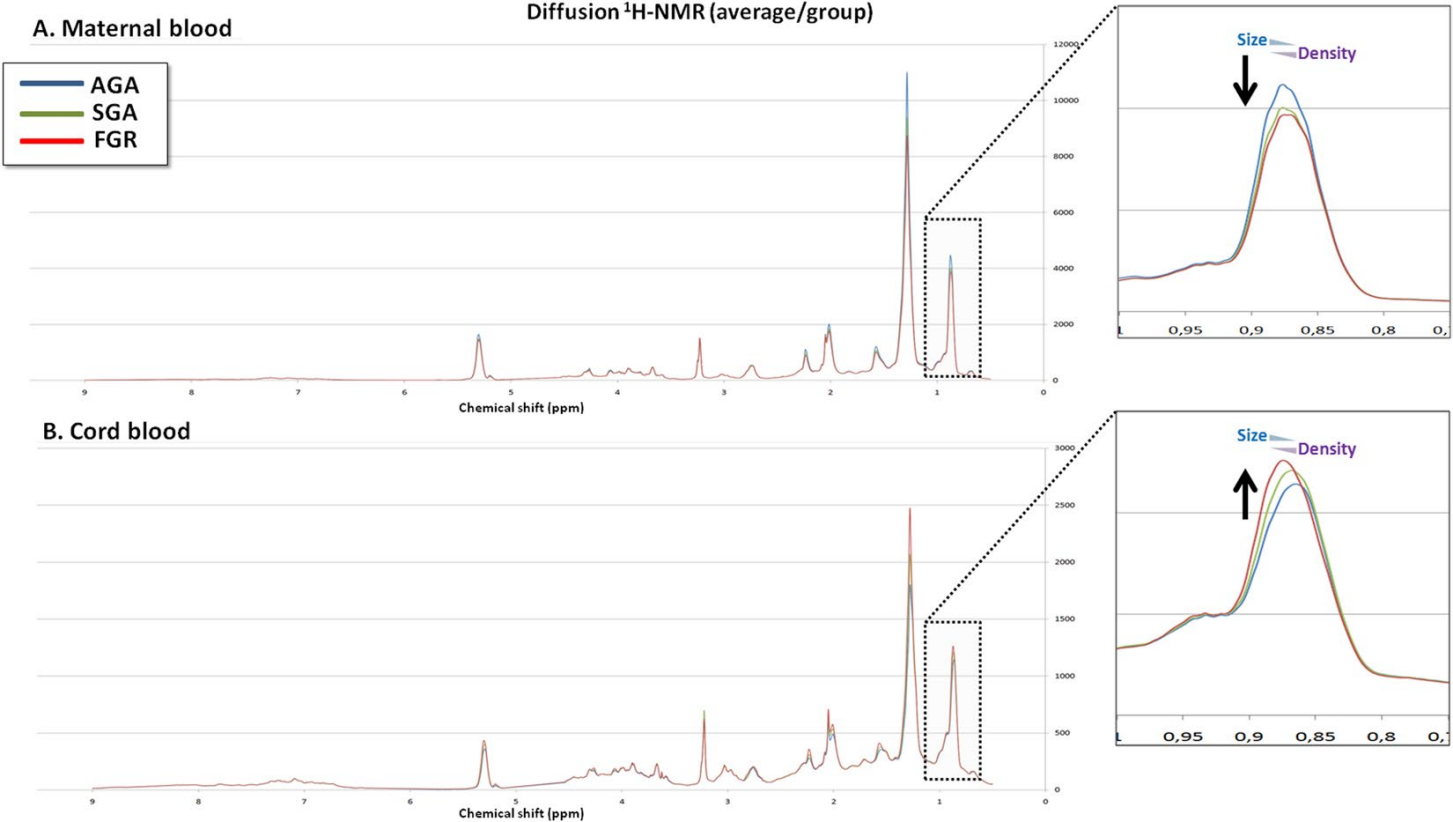
Maternal and cord blood nuclear magnetic resonance profiles among the study groups. The more evident differences in the *Diff* spectral vectors were observed at 1.29 and 0.89 ppm, suggesting lower relative plasma concentrations of lipids in mothers of small fetuses (both, in SGA and FGR cases), while in cord blood, the relative concentrations of lipids were higher in cases, being greater in FGR compared to SGA cases, as well as compared to controls.

**Figure 2:**
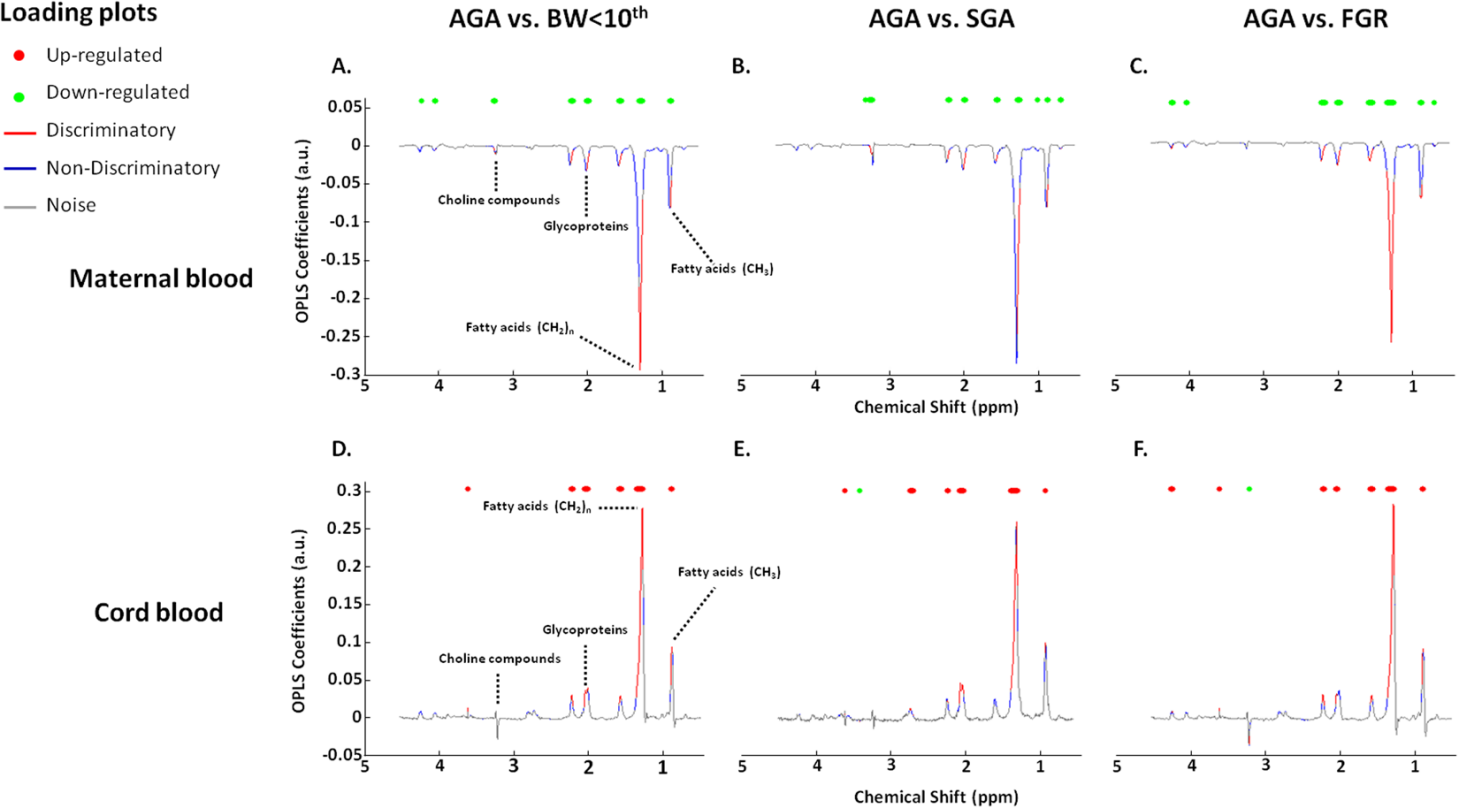
Loading weight of each spectral variable extracted from an orthogonal partial least square discriminant analysis model (ASCLAN) of the data set among the study population. The color-coded loading plot shows the discriminatory variables, non-discriminatory signal variables and the noise variables, which are indicated by red, blue and grey, respectively. The loading plot shows the most influential regions of the spectrum that are responsible for discrimination between cases and controls.

The average Diff spectral vectors generated for maternal and cord blood plasma are shown in Fig. 1. There were differences observed in the spectral vectors among the groups, more evident at 1.29 and 0.89 ppm, suggesting lower relative plasma concentrations of lipids in mothers of small fetuses (both, in SGA and FGR cases), while in cord blood, the relative concentrations of lipids were higher in cases, and mostly in the FGR group. To evaluate the ability of these metabolites in distinguishing small fetuses from controls, we performed non-targeted multivariate discriminant analysis. Fig. 2 represents the loading weight of each spectral point extracted from an OPLS-DA model of the data set (i.e. the most influential regions of the spectrum that are responsible for discrimination between cases and controls). The most discriminatory regions corresponded to lipids, CH_2_ and CH_3_, in both, mothers and fetuses [(CH_2_)_n_, 1.29 ppm and CH_3_ at 0.89 ppm)], confirming that these lipids: decrease in mothers of fetuses with FGR and SGA compared to controls, and increase in small fetuses. Furthermore, the approach yielded new findings such as an increase in choline compounds (in this case, most likely phosphatidylcholine) in fetuses (Fig. 2).

### Metabolic fingerprinting in clinical phenotypes of small fetuses

Based on the non-targeted metabolomics results obtained, quantitative targeted analyses using Liposcale (for lipid profiling, deconvolution of phosphatidylcholines and glycoproteins) and Dolphin (for LMW metabolite profiling) were performed (Fig. S1). The lipoproteins identified and their concentrations (i.e. triglycerides and cholesterol), strongly correlated to the enzymatic colorimetric methods quantifications for the same subjects (Fig. S2). Tables 2 and 3, shows the lipoproteins identified and their concentrations, phosphatidylcholines and glycoprotein properties, as well as LMW metabolites for each clinical phenotype (SGA and FGR) in maternal and cord blood, respectively. The Liposcale analysis showed that mothers of SGA fetuses had significantly lower concentrations of cholesterol-IDL compared to controls (p = 0.02). Similarly, triglycerides transporting lipoproteins, IDL and HDL, were significantly lower in mothers of fetuses with sub-optimal fetal growth compared to AGA mothers (p = 0.01, 0.03 and 0.02, respectively) (Table 2). In terms of lipoproteins properties, there were lower concentrations of large HDL lipoproteins in mothers of FGR fetuses (p = 0.04). Changes in glycoproteins and phosphatidylcholines suggested a disruption in their biophysical properties among study groups. Specifically, the width of one of the phosphatidylcholines peaks was significantly lower in FGR cases (p = 0.02). With regards to LMW metabolites, maternal plasma concentrations of alanine, citrate, 2-oxoisovaleric acid and pyruvate were significantly lower in mothers of small fetuses compared to controls (all p values < 0.05) (Table 2).

**Table 2:** Metabolic profiling and targeted metabolomics in maternal blood across the study groups.

		Adequate for Gestational Age N = 28	Small for Gestational Age N = 25	Fetal Growth Restriction N = 27	p values		
		Median (IQR)	Median (IQR)	Median (IQR)	AGA vs. SGA	AGA vs. FGR	Jonckheere-Terpstra test
**Lipids**							
Cholesterol (mg/dL)	VLDL	40.5 (30.7–68)	30.2 (23.9–49.7)	33.7 (24.3–49.8)	0.06	0.09	**0.04**
	IDL	35.4 (26.2–46.3)	30.9 (24.1–34.3)	30 (19.2–37.4)	**0.02**	0.06	**0.01**
	LDL	175 (150–217)	186.7 (170.2–197)	182.5 (148.6–201.5)	0.97	0.63	0.66
	HDL	78.9 (65.9–96.9)	80.9 (71–98.6)	86.5 (77.8–94.1)	0.75	0.24	0.89
Triglycerides (mg/dL)	VLDL	137.8 (109.8–228)	116.9 (87.6–163.9)	117.9 (92.5–170.2)	0.17	0.14	0.08
	IDL	30 (25.1–35.8)	26.1 (21.5–29.6)	27.4 (20–32.5)	**0.01**	0.09	**0.02**
	LDL	42.7 (36.1–52.1)	38.8 (32.2–42.8)	39.2 (27.9–44.3)	0.07	0.12	**0.04**
	HDL	36.8 (32.5–40)	30.5 (25.6–41.3)	32.3 (28.7–35.8)	**0.03**	**0.02**	**0.02**
Particle numbers (nmol/L) (umol/L)	VLDL						
	*Large*	3.2 (2.5–4.8)	3.1 (2.3–4.1)	2.9 (2.1–4.1)	0.3	0.14	0.08
	*Medium*	14.4 (11.3–21)	12 (8.5–16.7)	12.9 (10–16.5)	0.15	0.21	0.11
	*Small*	83.1 (65.2–146)	66.4 (50–99)	68 (52.9–104)	0.14	0.08	**0.05**
	LDL						
	*Large*	201 (163–249)	203 (170–223)	201 (152–217)	0.52	0.28	0.11
	*Medium*	520 (468–651)	527 (486–583)	526 (411–596)	0.98	0.44	0.28
	*Small*	621 (490–745)	591 (514–746.7)	632 (471–708)	0.90	0.61	0.30
	HDL						
	*Large*	0.72 (0.16–1.52)	0.24 (0.06–0.91)	0.37 (0.04–0.88)	0.08	**0.04**	**0.02**
	*Medium*	13.1 (10–17.7)	14 (12.9–17.1)	14.8 (13.2–16.7)	0.59	**0.04**	0.84
	*Small*	34.3 (30.1–39.8)	33.9 (28.4–38.7)	35.7 (32–38.4)	0.72	0.49	0.30
Phosphatidylcholines	Area peak 1	2 (1.6–2.7) × 10^6^	1.8 (1.6–2.2) × 10^6^	1.9 (1.2–2.4) × 10^6^	0.19	0.16	0.91
	Height peak 1	77 (62–101) × 10^3^	71 (67–79) × 10^3^	76 (51.7–91.7) × 10^3^	0.27	0.19	0.13
	Width peak 1	8.71 (8.3–9.1)	8.32 (7.7–9.2)	8.13 (7.7–8.8)	0.12	**0.02**	**0.01**
	Area peak 2	0.21 (0.1–0.3) × 10^6^	0.36 (0.14–0.5) × 10^6^	0.29 (0.1–0.4) × 10^6^	0.56	0.68	0.56
	Height peak 2	21 (9.5–28.4) × 10^3^	25 (14–35) × 10^3^	22 (10.7–31.4) × 10^3^	0.70	0.90	0.47
	Width peak 2	3.8 (3.2–4.3)	4.6 (3.3–4.9)	4.1 (3.5–4.6)	0.11	0.37	0.83
	Area peak 3	3.4 (2.3–4.1) × 10^6^	2.7 (1.7–4.2) × 10^6^	3 (2–4.1) × 10^6^	0.42	0.89	0.44
	Height peak 3	164 (105–226) × 10^3^	136 (80–198) × 10^3^	149 (97–220) × 10^3^	0.38	0.83	0.41
	Width peak 3	6.7 (6.4–7.2)	6.9 (6.5–7.2)	6.8 (6.5–7.2)	0.55	0.8	0.61
	Area peak 4	3.3 (2.8–3.9) × 10^6^	3.5 (2.7–4) × 10^6^	3.3 (3.1–3.9) × 10^6^	0.72	0.69	0.59
	Height peak 4	120 (103–151) × 10^3^	124 (108–144) × 10^3^	123 (111–137) × 10^3^	0.88	0.99	0.48
	Width peak 4	8.8 (8.4–9.2)	9.1 (8.5–9.6)	8.8 (8.3–9.4)	0.11	0.63	0.74
Glycoproteins	Area peak 1	0.47 (0.37–0.54) × 10^6^	0.49 (0.35–0.59) × 10^6^	0.5 (0.39–0.63) × 10^6^	0.83	0.18	0.89
	Height peak 1	23.4 (20–25.1) × 10^3^	23 (17–25.6) × 10^3^	23.5 (21–26.8) × 10^3^	0.76	0.51	0.71
	Width peak 1	6.8 (5.7–7.2)	6.80 (5.68–7.64)	7.16 (6.5–8.2)	0.48	0.07	**0.04**
	Area peak 2	2.8 (2.2–3.7) × 10^6^	2.4 (1.9–3.76) × 10^6^	2.7 (2.2–3) × 10^6^	0.39	0.44	0.27
	Height peak 2	124 (104–157) × 10^3^	110 (94–155) × 10^3^	123 (111–135) × 10^3^	0.38	0.48	0.70
	Width peak 2	7.37 (6.4–8.2)	6.88 (6.5–8.5)	7.28 (6.35–7.9)	0.8	0.4	0.28
	Area peak 3	30 (27–31.6) × 10^6^	25.6 (23.2–30.6) × 10^6^	26.7 (23.5–30.4) × 10^6^	0.09	0.08	**0.03**
	Height peak 3	280 (256–357) × 10^3^	261 (221–341) × 10^3^	248 (219–327) × 10^3^	0.07	0.13	**0.04**
	Width peak 3	35 (31–37.6)	36.3 (32.9–38.8)	34.3 (32.9–36.8)	0.24	0.53	0.66
**Low molecular weight metabolites (mM)**							
Acetate		0.05 (0.04–0.08)	0.06 (0.05–0.12)	0.07 (0.05–0.09)	0.07	0.19	0.07
Acetone		0.22 (0.13–0.31)	0.19 (0.13–0.25)	0.21 (0.11–0.27)	0.67	0.29	0.16
Alanine		1.17 (1.02–1.34)	1.15 (0.99–1.34)	1.02 (0.89–1.19)	0.55	**0.04**	**0.02**
Citrate		0.69 (0.64–0.74)	0.64 (0.56–0.69)	0.56 (0.50–0.68)	**0.04**	**0.01**	**0.002**
Creatine		0.13 (0.11–0.15)	0.12 (0.09–0.16)	0.13 (0.10–0.16)	0.95	0.63	0.67
Creatinine		0.20 (0.18–0.22)	0.2 (0.17–0.22)	0.19 (0.16–0.21)	0.73	0.35	0.17
Formate		0.09 (0.07–0.11)	0.1 (0.09–0.12)	0.09 (0.08–0.13)	0.17	0.48	0.78
Glucose		5.58 (4.90–6.19)	5.8 (4.13–6.89)	5.35 (4.93–6.39)	0.83	0.93	0.54
Glutamine		1.1 (0.9–1.2)	1.09 (0.80–1.27)	1.06 (1.0–1.33)	0.95	0.51	0.76
Glycine		0.59 (0.48–0.66)	0.58 (0.51–0.66)	0.64 (0.51–0.73)	0.64	0.39	0.78
Histidine		0.24 (0.20–0.28)	0.20 (0.17–0.27)	0.22 (0.19–0.24)	0.33	0.17	0.11
Isoleucine		0.17 (0.15–0.2)	0.15 (0.12–0.2)	0.16 (0.13–0.2)	0.14	0.5	0.26
Lactate		11 (7.57–14)	11.67 (7.14–15.2)	11 (7.16–13.5)	0.98	0.61	0.32
Leucine		0.22 (0.2–0.26)	0.21 (0.16–0.25)	0.24 (0.18–0.28)	0.26	0.83	0.56
Mannose		0.21 (0.19–0.23)	0.2 (0.16–0.23)	0.19 (0.17–0.2)	0.16	0.13	0.06
2-oxoisovaleric acid		1.02 (0.84–1.27)	0.77 (0.66–0.94)	0.78 (0.69–0.94)	**0.0009**	**0.002**	**0.001**
Phenylalanine		0.28 (0.26–0.3)	0.3 (0.26–0.32)	0.3 (0.27–0.35)	0.22	0.1	**0.04**
Pyruvate		0.65 (0.43–0.75)	0.5 (0.34–0.73)	0.44 (0.35–0.56)	0.18	**0.05**	**0.03**
Tyrosine		0.32 (0.28–0.37)	0.32 (0.28–0.37)	0.32 (0.27–0.37)	0.8	0.90	0.43
Valine		0.52 (0.45–0.57)	0.51 (0.42–0.59)	0.52 (0.46–0.57)	0.58	0.81	0.57

HDL: High-density lipoprotein; IDL: Intermediate density lipoprotein; IQR: Interquartile range; LDL: Low-density lipoprotein; VLDL: Very low-density lipoprotein. Concentration, size and properties of the peaks are presented as median and interquartile range (IQR).

**Table 3:** Metabolic profiling and targeted metabolomics in cord blood across the study groups.

		Adequate for Gestational Age N = 28	Small for Gestational Age N = 25	Fetal Growth Restriction N = 27	p values		
		Median (IQR)	Median (IQR)	Median (IQR)	AGA vs. SGA	AGA vs. FGR	Jonckheere-Terpstra test
**Lipids**							
Cholesterol (mg/dL)	VLDL	8.6 (7.47–11.1)	10.8 (8.4–13.7)	13.5 (9.2–17.2)	0.1	**0.008**	**0.002**
	IDL	7.1 (6.4–8.9)	8.6 (7.2–10.5)	8.8 (8–10.8)	0.08	**0.04**	**0.01**
	LDL	82.1 (79–86)	80.7 (78.9–86.5)	80.5 (78.3–86.9)	0.98	0.73	0.63
	HDL	61.9 (58.9–65.1)	61.6 (58.7–64.2)	56.5 (53.5–63.3)	0.81	0.06	0.97
Triglycerides (mg/dL)	VLDL	39.6 (35.7–45.4)	45.3 (37.8–52.3)	50.4 (42.2–61.7)	0.1	**0.007**	**0.002**
	IDL	7.8 (7.1–9.4)	8.9 (7.9–10.5)	9.3 (8.6–11.1)	0.08	**0.03**	**0.008**
	LDL	6.7 (5.8–8.5)	8.1 (6.2–9.3)	7.3 (6.7–9.7)	0.2	0.09	0.04
	HDL	12.4 (11.3–13.4)	12.7 (12.1–13.1)	12.3 (11.6–13)	0.22	0.92	0.44
Particle numbers (nmol/L) (umol/L)	VLDL						
	*Large*	0.9 (0.75–1.1)	1.1 (0.79–1.32)	1.23 (1–1.6)	0.15	**0.004**	**0.001**
	*Medium*	4.8 (4.5–5.6)	5.57 (4.7–5.9)	6 (5–6.8)	0.11	**0.003**	**0.001**
	*Small*	19.6 (16.5–24.3)	20.6 (17.1–30.6)	27.5 (19.4–34.3)	0.22	**0.03**	**0.01**
	LDL						
	*Large*	82.9 (74–88.7)	83.7 (78.4–91)	83.5 (73–90.6)	0.76	0.86	0.56
	*Medium*	188 (180–202)	201 (181–209)	202 (192–213)	0.13	**0.04**	**0.02**
	*Small*	294 (270–315)	286 (267–329)	292 (257–317)	0.55	0.67	0.74
	HDL						
	*Large*	0.23 (0.14–0.31)	0.23 (0.18–0.35)	0.34 (0.26–0.5)	0.7	**0.009**	**0.003**
	*Medium*	10.4 (9.6–11.7)	10.8 (10.2–11.8)	9.7 (7.4–11.7)	0.48	0.06	0.1
	*Small*	19.6 (17.9–20.8)	18.9 (17.6–22)	18.9 (17.7–21.1)	0.78	0.59	0.72
Phosphatidylcholines	Area peak 1	0.4 (0.32–0.44) × 10^6^	0.42 (0.32–0.54) × 10^6^	0.51 (0.4–0.58) × 10^6^	0.32	**0.04**	**0.01**
	Height peak 1	14.5 (13.2–16.5) × 10^3^	16.5 (13–19) × 10^3^	17.6 (15.5–21.4) × 10^3^	0.18	**0.006**	**0.003**
	Width peak 1	8.9 (8.1–9.6)	8.6 (8.3–9.2)	8.5 (8.2–9.4)	0.34	0.37	0.86
	Area peak 2	0.34 (0.2–0.41) × 10^6^	0.41 (0.21–0.5) × 10^6^	0.26 (0.15–0.48) × 10^6^	0.22	0.42	0.72
	Height peak 2	17 (13.6–20.4) × 10^3^	20 (14.4–26) × 10^3^	16 (10–24.2) × 10^3^	0.09	0.88	0.36
	Width peak 2	6.37 (4.6–7.3)	6.5 (5.4–6.9)	5.7 (4.5–7.1)	0.98	0.19	0.94
	Area peak 3	0.79 (0.34–1.18) × 10^6^	1.19 (0.53–1.83) × 10^6^	0.66 (0.22–1.56) × 10^6^	0.31	0.78	0.57
	Height peak 3	35.1 (21.4–53.2) × 10^3^	55.2 (27.4–78.4) × 10^3^	31.7 (12.6–59) × 10^3^	0.21	0.76	0.58
	Width peak 3	7 (5.8–8.1)	7.1 (5.3–7.9)	7.4 (5.8–9.2)	0.62	0.59	0.26
	Area peak 4	2.1 (1.85–2.41) × 10^6^	1.76 (1.1–2.42) × 10^6^	1.76 (1.3–2.29) × 10^6^	0.12	0.05	0.03
	Height peak 4	76 (65.2–87.8) × 10^3^	62.3 (46.3–82.3) × 10^3^	64.7 (49.1–81) × 10^3^	0.08	0.07	**0.05**
	Width peak 4	9.3 (8.74–9.6)	9.1 (8.5–9.5)	8.9 (8.4–9.2)	0.26	0.07	0.04
Glycoproteins	Area peak 1	0.44 (0.36–0.56) × 10^6^	0.43 (0.36–0.56) × 10^6^	0.46 (0.37–0.56) × 10^6^	0.96	0.71	0.37
	Height peak 1	13 (11.8–14.4) × 10^3^	12.7 (11.4–14.3) × 10^3^	13.9 (11.8–16.1) × 10^3^	0.53	0.21	0.14
	Width peak 1	11.3 (10.2–12.6)	10.9 (10–12)	11 (10.1–12.1)	0.43	0.5	0.72
	Area peak 2	1.3 (1.2–1.4) × 10^6^	1.4 (1.3–1.69) × 10^6^	1.6 (1.4–2) × 10^6^	0.08	**0.002**	**0.0004**
	Height peak 2	52.4 (48–56.2) × 10^3^	53 (50–66) × 10^3^	59.9 (53.5–70.2) × 10^3^	0.17	**0.009**	**0.003**
	Width peak 2	8.3 (7.9–8.5)	8.4 (8.1–8.6)	8.6 (8.4–9.2)	0.24	**0.005**	**0.002**
	Area peak 3	7.2 (5.6–8.4) × 10^6^	7.3 (6.3–8.9) × 10^6^	6.7 (5.5–7.7) × 10^6^	0.59	0.64	0.68
	Height peak 3	55 (44–64) × 10^3^	61.2 (48–71) × 10^3^	59 (51.4–70.4) × 10^3^	0.15	0.19	0.1
	Width peak 3	46.6 (38–50)	43.7 (37–49.8)	38 (33.2–43.9)	0.18	**0.002**	**0.0005**
**Low molecular weight metabolites (mM)**							
Acetate		0.06 (0.05–0.08)	0.07 (0.06–0.09)	0.08 (0.07–0.10)	0.07	**0.01**	**0.003**
Acetone		0.12 (0.09–0.23)	0.12 (0.08–0.21)	0.1 (0.07–0.17)	0.69	0.24	0.87
Alanine		1.7 (1.41–1.9)	1.6 (1.4–1.9)	1.7 (1.4–1.9)	0.66	0.90	0.47
Citrate		0.53 (0.5–0.6)	0.6 (0.5–0.6)	0.6 (0.4–0.61)	0.96	0.6	0.67
Creatine		0.16 (0.12–0.2)	0.17 (0.13–0.2)	0.18 (0.15–0.25)	0.72	0.25	0.12
Creatinine		0.17 (0.16–0.2)	0.18 (0.17–0.2)	0.18 (0.14–0.22)	0.34	0.78	0.33
Formate		0.12 (0.1–0.15)	0.16 (0.12–0.17)	0.16 (0.11–0.19)	**0.02**	**0.02**	**0.004**
Glucose		4.25 (3.88–5.1)	4.42 (3.7–5.03)	4.6 (3.9–5.38)	0.84	0.33	0.2
Glutamine		1.35 (1.2–1.5)	1.39 (1.29–1.6)	1.42 (1.24–1.56)	0.42	0.53	0.26
Glycine		1.1 (0.96–1.22)	1.1 (0.94–1.1)	1.1 (0.97–1.22)	0.64	0.63	0.3
Histidine		0.42 (0.39–0.45)	0.39 (0.34–0.44)	0.39 (0.35–0.46)	0.22	0.2	0.09
Isoleucine		0.23 (0.19–0.26)	0.2 (0.18–0.25)	0.23 (0.2–0.26)	0.17	0.96	0.56
Lactate		16.9 (13.2–21.4)	16.1 (13.5–23.6)	18.7 (12.2–25.2)	0.99	0.61	0.35
Leucine		0.35 (0.32–0.42)	0.35 (0.3–0.4)	0.36 (0.31–0.42)	0.53	0.98	0.52
Mannose		0.96 (0.79–1.1)	0.89 (0.78–0.96)	0.89 (0.8–1.03)	0.2	0.72	0.68
2-oxoisovaleric acid		0.1 (0.08–0.11)	0.1 (0.08–0.12)	0.11 (0.09–0.12)	0.65	0.14	0.07
Phenylalanine		1.46 (1.05–1.64)	1.20 (0.89–1.5)	1.26 (0.91–1.42)	0.75	0.13	0.23
Pyruvate		0.46 (0.44–0.49)	0.48 (0.46–0.53)	0.52 (0.44–0.56)	0.26	0.2	0.08
Tyrosine		0.71 (0.6–0.89)	0.6 (0.4–0.94)	0.84 (0.54–0.91)	0.27	0.99	0.55
Valine		1.12 (0.92–1.32)	1.1 (0.95–1.35)	1.18 (0.92–1.41)	0.99	0.74	0.4

HDL: High-density lipoprotein; IDL: Intermediate density lipoprotein; IQR: Interquartile range; LDL: Low-density lipoprotein; VLDL: Very low-density lipoprotein. Concentration, size and properties of the peaks are presented as median and interquartile range (IQR).

**Figure 3:**
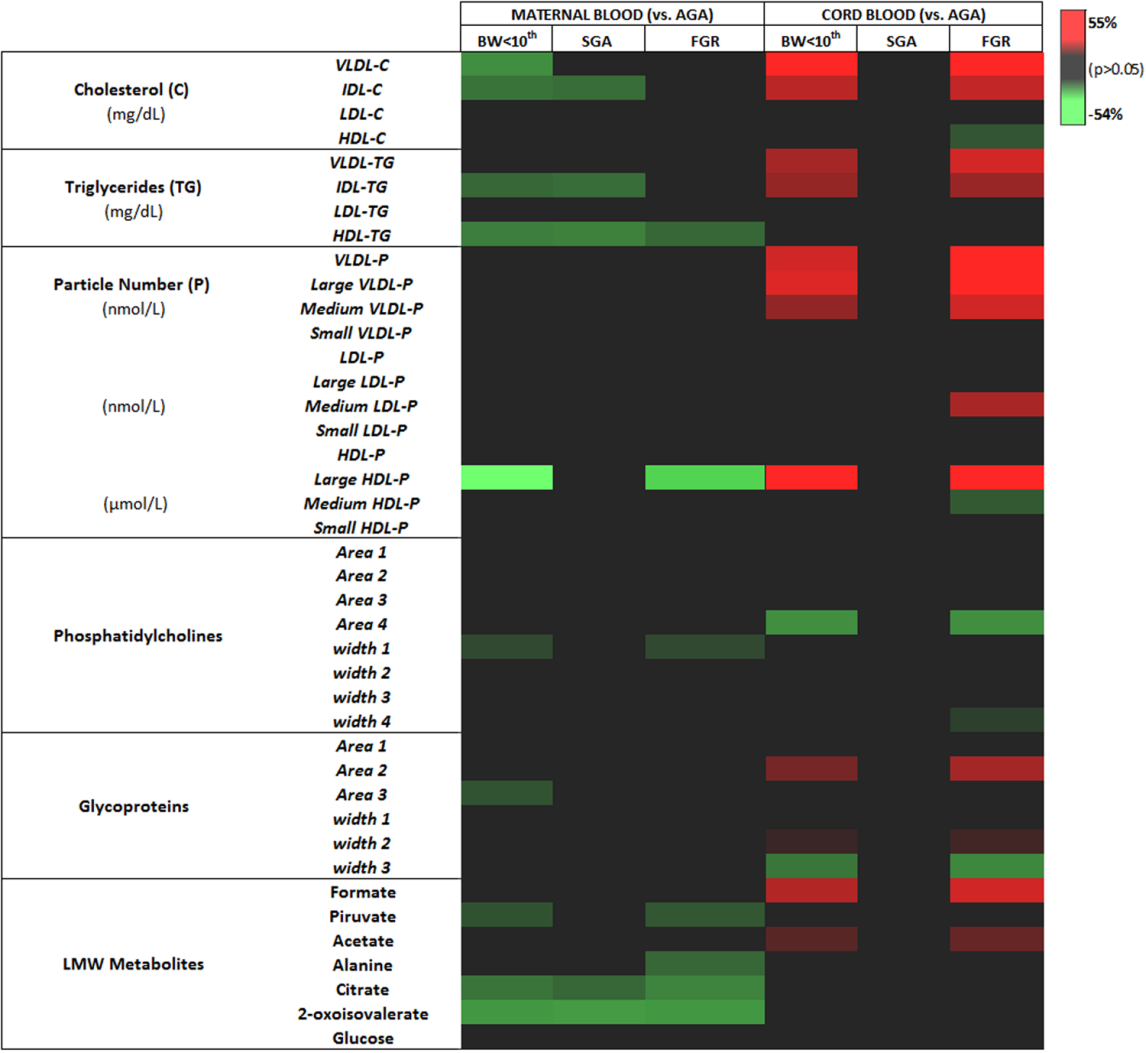
Heat map following metabolomic profiling on the annotated and significant (among the three groups) identified in plasma samples of mothers and neonates. A colored heat map of significant LMW metabolites and the Liposcale analysis along with their p-values, and percentage of fold change was obtained to visualize and characterize the differences between adequate for gestational age (AGA), all cases with a birthweight below the 10^th^ centile (BW <10^th^) and separately small-for-gestational age (SGA) as well as fetal growth restriction (FGR) groups from maternal and umbilical cord blood. Fold change of significant differences (p < 0.05) for each metabolite are on a graded color scale from green (lower value) to black (no differences) to red (high values).

Interestingly, the behavior of triglycerides and cholesterol lipoproteins was the opposite in affected fetuses compared to their mothers. FGR fetuses had significantly higher concentrations of cholesterol-VLDL (+56%), -IDL (+24%) lipoproteins (p = 0.008 and 0.04, respectively), as well as higher concentrations of triglycerides-VLDL (+24%), and -IDL (+18%) compared to controls (p = 0.008 and 0.04, respectively) (Table 3). Importantly, we found a gradient of higher concentrations of cholesterol (VLDL and IDL) and triglycerides (VLDL, IDL and LDL) lipoproteins in SGA and FGR cases compared to controls (Jonckheere–Terpstra test p < 0.05). FGR cases also showed a significant increase in large HDL, medium LDL and all VLDL particle types (large, medium and small: average + 37%) vs. controls (all p values < 0.05). When analyzed as a trend, the Jonckheere-Terpstra test also showed that the increase in particles size and distribution was present in the two clinical phenotypes following a severity ascendant gradient, higher in FGR fetuses. Likewise, changes in phosphatidylcholines and glycoproteins peaks were more prominent in FGR fetuses vs. controls (Table 3). Conversely, among LMW metabolites, formate and acetate showed a significant trend towards increase in cord blood of FGR fetuses (p = 0.02 and 0.01, respectively) (Table 3). No differences were found in the concentrations of glucose between the study groups. Figure. 3 is a heat map constructed to visualize and summarize the significant differences on the maternal and cord blood plasma LMW metabolites, lipid classes, particles and subclasses along with their p-values, and percentage of fold change (among cases and controls).

## Discussion

### Principal findings of the study

In this comprehensive metabolomic study, we expanded previous observations that lipid profiles are disturbed in small fetuses and their mothers. Yet, our innovative approach allowed quantifying the number and size of the particles in the lipoprotein fractions as well as low-molecular-weight metabolites, describing the complex maternal and fetal metabolic response in pregnancies with fetal smallness. However, the study failed to observe any remarkable difference between the two clinical phenotypes of fetal smallness. Thus, SGA and FGR presented similar findings, supporting the notion that both phenotypes might be exposed to different degrees of undernutrition and thus, both might be at risk of metabolic fetal programming. Future studies assessing placenta metabolism might delineate potential mechanisms in both phenotypes.

### Why are circulating lipids lower in mothers of growth-restricted fetuses?

Our results describing an overall decrease in circulating lipids in mothers of small fetuses are in line with previous studies^42–47^. However, this study revealed further details, providing qualitative and quantitative measurements of lipoproteins, their cholesterol and triglyceride concentration, as well as the number of sub-fraction particles and their size. Compared to controls, there was a triglyceride-impoverishment in lipoproteins such as HDL (decrease in large particles) and IDL as well as low concentration of cholesterol in other lipoproteins, namely VLDL and IDL in mothers of both SGA and FGR cases. A maternal metabolic adaptation during pregnancy^47–53^ is essential to increase availability of cholesterol and triglycerides for fetal development and the synthesis of steroid hormones by the placenta^54–56^. When maternal plasma cholesterol is low, birthweight is lower than normal. Indeed, term infants of mothers with low total cholesterol weigh are on average 150 g less than those born to mothers with normal cholesterol concentrations^57^. The first trimester of gestation is considered an anabolic period, in which the mother increases her deposit of fat, through a reduction in the activity of *lipoprotein lipase*, and increasing in the insulin responsiveness^58^. Interestingly, this fat accumulation is maintained even under conditions of severe maternal malnutrition^59^. In the third trimester, a switch to catabolism occurs in order to supply fetal demands and to synthesize large amounts of steroids hormones by the placenta^54^. The products of an enhanced lipolytic activity reach the liver to be used for triglycerides synthesis and to increase circulating availability of triglycerides by 208% (transported mainly by VLDL), and total, HDL- and LDL-cholesterol in 65, 26 and 64%, respectively^50^. It seems plausible that in the third trimester of pregnancy, placental insufficiency generates a specific fetal and maternal response that is reflected in the metabolomic profile. Low concentrations of lipids in the maternal circulation may reflect an increased uptake into the placenta. In normal gestation, support of lipids, and particularly essential fatty acids, to the fetus is ensured by increased maternal liver production of VLDL rich in triglycerides (build upon peripheral deposits of adipose tissue)^60^, peripheral enrichment of HDL and LDL and lipoprotein lipase activities in the placenta^61,62^. The findings of this and previous studies suggest (1) failure of the normal signaling leading to increased lipids in pregnancy, and/or (2) a reduction in lipid reserve in the mothers.

### Why are there higher cord blood lipids in SGA and FGR fetuses?

Our findings confirmed previous studies reporting higher circulating triglycerides and cholesterol lipoproteins in growth-restricted fetuses^63–66^. Importantly, our metabolic fingerprinting approach allowed to describe that VLDL and IDL were the most deregulated lipoproteins in FGR fetuses, suggesting that VLDL rich in triglycerides may be an alternative fuel mobilized by the growth-restricted fetus. This is a novel finding in humans, since VLDL is mostly synthesized in the fetal liver, implying an altered hepatic synthesis of lipoproteins in adverse intrauterine conditions.

Lipids are fundamental molecules for life. During fetal life, triacylglycerols provide energy for metabolic processes, while fatty acids, cholesterol and phospholipids are required to develop the fetal brain and central nervous system, to build cell membranes, and as a precursor of bile acids and steroid hormones^67^. Both, the placenta and the fetus have the capacity of *denovo* cholesterol synthesis. It has been estimated that up to 20% of cholesterol could derive from transplacental passage. Maternal cholesterol-carrying lipoproteins (mainly HDL) are transported across trophoblast and then overpass the endothelial cells of the fetoplacental vasculature, to finally being effluxed into the fetal circulation^60^. Thus, like in the adult, the liver is the principal source of circulating lipoproteins in the fetus. Previous animal^68^ and human studies have shown higher concentrations of circulating triglycerides^64,69,70^, as well as an altered, pro-atherogenic lipid and cholesterol metabolism in growth-restricted fetuses^38,71,72^. The lipid profile of FGR fetuses described herein is akin to adults with dyslipidemia and atherosclerosis. Large epidemiological as well as experimental evidence have suggested that an adverse intrauterine environment leading to low birthweight may increase the risk for cardiovascular disease later in life^73–77^. Therefore, important challenges for future research are to ascertain the mechanistic pathways whereby fetal abnormal lipid profile might influence the higher risk of growth restricted fetuses to cardiovascular disease in adulthood.

### Changes in low molecular weight metabolites

While major changes were found in lipids, analysis of LMW metabolites revealed lower cord blood concentrations of acetate and formate in late-onset FGR and maternal concentrations of citrate, pyruvate and 2-oxoisovaleric acid in both, SGA and FGR pregnancies. Biophysical abnormalities in phosphatidylcholines and glycoproteins were also detected. Phosphatidylcholine is a major membrane phospholipid, made in mammalian cells from choline via the CDP-choline pathway and has a key role in neuronal differentiation and cell fate determination^78^. Importantly, phosphatidylcholine biosynthesis is required for normal VLDL secretion from hepatocytes^79^. Metabolomic analysis showed some differences in comparison with previous studies. For instance, in this study we did not replicate our previous finding of lower concentrations of valine and leucine in late-onset FGR^38^, nor we could demonstrate differences in other essentials aminoacids in SGA fetuses as reported in other studies^36,37,80^. Favretto *et al*. reported up regulation of phenylalanine, tryptophan, and glutamate in FGR fetuses^37^, while Ivorra *et al*. reported significant differences cord blood concentrations of five aminoacids in FGR fetuses (proline, glutamine and alanine were reduced, while phenylalanine and citrulline were increased)^36^. In previous studies, early-onset FGR showed significant decrease in glucose^63,81^, while no differences have been found in late-onset FGR^63^. It is known that glucose levels are inversely correlated to the clinical severity of FGR and the capability of transplacental glucose gradient, which might differ between the two phenotypes. Thus, these discrepancies suggest differences in case selection, platforms and protocols used among studies assessing metabolites in placental dyfunction^36,37^.

### Metabolic differences between small-for-gestational age and growth-restricted fetuses

FGR has two main clinical presentations, early-and late-onset disease. Early-onset FGR is highly associated with severe placental insufficiency and chronic fetal hypoxia^82,83^. In early-onset FGR, the typical scenario is a progressive deterioration of fetal well-being, accompanied with higher rates of preeclampsia and adverse perinatal outcomes, often requiring preterm delivery^4^. On the other hand, late-onset FGR represents 70–80% of FGR, has less signs of placental disease and the association with preeclampsia is minimal^84^. Despite a more benign nature as compared with early FGR, and that chronic hypoxia seems to be mild, the increase demands of oxygen and nutrients by vital organs such as brain and heart towards term increase the risk of acute fetal deterioration before labor, as suggested by the high contribution to late-pregnancy mortality, and a high association with intra- partum fetal distress and neonatal acidosis^85^. One of the aims was to evaluate whether late-onset SGA and FGR are associated with differential metabolomic patterns. Although there is a widely shared assumption that SGA are “constitutionally small”, but otherwise normal, fetuses, more recent studies reported that SGA display similar, albeit milder, neurodevelopmental and cardiovascular changes as those observed in FGR fetuses^13,14,25,86,87^. In line with this, the present study found that SGA and FGR fetuses had remarkably similar pattern of alterations in maternal and fetal lipid profiles, although there was a gradation of metabolic disruption according to the severity of the disease. These findings support that, at least a proportion of SGA fetuses represent a milder clinical form of true growth restriction not associated with Doppler changes or increased obstetrical risks. It could be hypothesized that SGA fetuses suffer undernutrition and its long-term consequences, but the respiratory function of the placenta is still largely preserved. Consequently, while the clinical distinction between “low risk” SGA and FGR is relevant for obstetrical management, fetal smallness represents a (potential) high-risk situation for long-term quality of life since metabolic adaptations occurs in utero regardless of its clinical prenatal presentation.

### Strengths and limitations

The strengths of this study include the prospective design and the inclusion of a well-defined cohort including samples from mothers and fetuses of both, SGA and FGR cases with a relatively large sample size, meaning that the possibility of selection bias was minimized. From a methodological standpoint, the platforms used are highly reproducible and standardized. NMR and mass spectrometry are not mutually exclusive but rather complementary. They have advantages and disadvantages, and the method of choice depends on the aim of the study. One of the main advantages of NMR is its extraordinary reproducibility, which makes this technique a very good option for fingerprinting analysis, such as the study reported herein. Besides identification, we had the interest of quantify specific lipids, therefore we have used NMR spectroscopy since the peak area of a compound in the NMR spectrum is directly related to the concentration of specific nuclei, making quantification of compounds in a complex mixture very precise^88,89^. On the other hand, NMR is a non-destructive technique that allows samples to be recovered after analysis, which is not trivial considering the incalculable value of these samples. While basal contamination with macromolecules and lipids in the plasma are usually overlooked, herein, were discriminated and quantified and in addition, the resolution of spectral process described (buckets within 0.003 ppm) is significantly higher to that reported before (buckets within 0.05 ppm)^36–38^. Moreover, the meticulous deconvolution process applied to each metabolite, is by far more rigorous, than the “blinded” quantification previously used^36–38^.

This is the first time that Liposcale is used in pregnancy. This test was originally developed to rapidly produce a lipoprotein profile of an individual’s plasma as part of cardiovascular disease risk work up^90,91^. The positive correlations describe between Liposcale and enzymatic colorimetric methods suggests that these variations are biologically relevant. Yet, we acknowledge several limitations. First, our sample size may cover potential associations in other metabolites and hide interaction of confounders (clinical characteristics similar among populations but non-obvious confounders could have affected). Secondly, as we focused in late-onset FGR (by far, the most common clinical presentation), we might have missed the most extreme and severe cases occurring before 32 weeks of gestation, in which fetal metabolic adaptations might be of uttermost relevance. Third, it is likely that our classification has overlapping between SGA and FGR, and for instance it is likely that constitutional small fetuses were included. Fourth, because of the design of the study, we only had few maternal samples of elective cesarean sections (maternal fasting conditions) and thus, non-fasting blood samples were collected. Typically, there is marginal effect of fasting on total cholesterol, HDL-C, and LDL-C, although triglycerides have a tendency to increase approximately 15% in non-fasting vs. fasting samples^92–94^. Fifth, although questionable, other more difficult to control confounding factors such as differences in the length of labor, time lapse difference between maternal and fetal sampling^95^ or seasonal variation of lipid profile^96^ might influence maternal lipid profile in the same form as fetal gender might influence the metabolomic profile of the fetuses included^97^. Future studies with a larger sample size might include these confounding factors into the analysis.

## Conclusion

In summary, metabolic profiling combined with clinical phenotyping demonstrates the potential to enrich stratified medicine research, revealing that mothers of small fetuses present substantial reductions in lipid metabolites, suggesting a failure in the maternal metabolic adaptation to pregnancy. While, both, SGA and growth-restricted fetuses have a substantial increase in lipids, indicating a similar metabolic response to undernutrition, there was a gradation of the metabolic disruption according to the severity of the phenotype.

## Materials and Methods

### Subjects and sample collection

This study is part of a larger prospective research program on FGR at the Department of Maternal-Fetal Medicine in Hospital Clinic Barcelona, involving maternal, fetal, and perinatal outcomes, as well as short- and long-term postnatal follow-up. A prospective cohort of singleton gestations with antenatal suspicion of fetal smallness and diagnosis established later than 32 weeks of gestation, were prospectively followed; those who delivered neonates with a birthweight below the 10^th^ centile at term (≥37 weeks of gestation) were included as cases (n = 52). According to our clinical protocol, small fetuses were subdivided: those with a birthweight <3^rd^ centile and/or abnormal uterine artery Doppler and/or abnormal cerebroplacental ratio were termed *fetal growth restriction* (FGR), while those with a birthweight between the 3^rd^ and the 9^th^ centile and normal fetoplacental Doppler were considered *small-for-gestational age* (SGA) cases. A control group of 28 pregnancies with adequate-for-gestational ages (AGA) fetuses were selected among low-risk pregnancies attending third trimester routine pregnancy care and were included if they delivered term neonates with a normal birthweight (between 20^th^ and 90^th^ centile). Exclusion criteria included multiple gestations, spontaneous preterm labor or delivery, premature rupture of membranes, chromosomal abnormalities or major structural abnormalities. All the patients included in this study were delivered at term and did not receive steroids for fetal lung maturity. The Institutional Research and Ethics Committee approved the study protocol (review board 2014/7154), all parents gave their written informed consent and all experiments were performed in accordance with relevant guidelines and regulations.

### Clinical and Ultrasound Data

Maternal, perinatal and neonatal data were prospectively recorded in all patients. Gestational age was calculated on the basis of fetal crown-rump length, measured at 11–13 weeks. Transabdominal ultrasound with Doppler evaluation was performed in both, cases and controls, at recruitment, using 6-4-MHz probes (Siemens Sonoline Antares, Siemens Medical Systems, Malvern, PA, USA) and a Voluson 730 Expert Machine (GE Medical systems, Zipf, Austria). Estimated fetal weight (EFW) was calculated using the Hadlock formula^98^, and adjusted according to fetal gender and gestational age using local standards^99^. Feto-placental Doppler included: umbilical artery-pulsatility index (PI), mean uterine artery PI and middle cerebral artery PI. The cerebroplacental ratio was calculated as middle cerebral artery PI/umbilical artery PI^100^, and defined as abnormal when <5^th^ centile for gestational age^100^. The mean uterine arteries PI value was considered abnormal when >95^th^ centile^101^. Cases of FGR were delivered electively between 37 and 38 weeks of gestation, while SGA cases were allowed to deliver up to 40 weeks of gestation^5,6,11^. Controls were allowed to have a spontaneous onset of labor but, if they reached 41 weeks of gestation, induction of labor was offered.

### Biological samples collection and storage

Maternal blood samples were drawn 2–4 hours after delivery (non-fasting conditions but at least eight hours after their last meal and blood sampling). Umbilical vein cord blood samples were obtained from the clamped umbilical cord immediately after delivery of the fetus. All blood samples were collected in EDTA-treated tubes and processed within one hour. Plasma was separated by centrifugation at 3000 rpm for 10 minutes at 4 °C, and stored at −80 °C until further use.

### NMR data acquisition

Plasma samples were thawed overnight and prepared for nuclear magnetic resonance (NMR) analyses according to the Bruker-specific metabolomics protocol^102^. Aliquots of each sample (300 μl) were mixed with 300 μl of sodium phosphate buffer for immediate analysis. High-resolution ^1^H-NMR spectroscopy data were acquired on a Bruker 600 MHz Spectrometer (Bruker Biospin, Rheinstetten, Germany) equipped with an Avance III console and a TCI CryoProbe Prodigy: 1D Nuclear Overhauser Effect SpectroscopY (NOESY), Carr-Purcell-Meiboom-Gill (CPMG), and 2D j-resolved spectroscopy (JRES), all with pre-saturation to suppress the residual water peak, to characterize small molecules such as amino acids and sugars; and 1D Diffusion (Diff, TR/TE, b values, NS, ACQ), to detect larger molecules such as lipoproteins, glycoproteins and choline compounds^103,104^. All the sequences were run at 37 °C in quantitative conditions (systematic pre-calibration of radio frequency pulses and sample temperature, and same receiver gain adjustment). CPMG and Diff data were preprocessed at the NMR console (TopSpin 3.2, Bruker Biospin, Rheinstetten, Germany) for basic corrections, such as phase correction and exponential line broadening (0.5 Hz for CPMG; 1.0 Hz for Diff).

### NMR fingerprinting analysis

For the metabolic fingerprinting analysis, spectral vectors were generated from the CPMG and Diff data by binning (0.003 ppm), alignment (alanine, 1,475 ppm) and region suppression (to minimize the influence of water and EDTA signals, specific to the CPMG data). This was carried out with MestReNova v11.0.3 (Mestrelab Research S.L., Santiago de Compostela, Spain). These spectral vectors were used for automatic spectroscopic data categorization by clustering analysis (ASCLAN), a supervised method to quickly assess discriminatory spectral regions (and indirectly metabolites) in the study groups^105^. The Diff NMR data was further used for lipoprotein profiling, based on the Liposcale test^90,91^. This test provides information about the size, lipid concentration (cholesterol and triglycerides) and number of particles for the main classes of lipoproteins [very-low density lipoprotein (VLDL), low-density lipoprotein (LDL), intermediate-density lipoprotein (IDL) and high-density lipoprotein (HDL)], as well as the concentration of particles in their subclasses (large, median, small)^90,91^. The Diff NMR data was also used to quantify choline compounds (3.3–3.18 ppm) and glycoproteins (2.15-1.9 ppm), based on peak deconvolutions. Details of the method for the Liposcale for lipoprotein characterization and phosphatidylcholines compounds and glycoprotein peak deconvolution are explained in the supplemental material. Finally, CPMG data was used for the profiling of 20 metabolites, based on a new, fully automated version of the software package Dolphin^106,107^. Signal annotation was based on templates prepared in previous studies with the help of available databases^108^ and bibliography^109–111^. Validation of metabolite identification was assisted by STOCSY^112^ and JRES data.

### Statistical analysis

Categorical data are presented as n (%) and continuous data as mean (±SD) or median [interquartile range (IQR)] according to their distribution. To assess the categorical variables, proportions were compared with Fisher’s exact test or the chi-square test. Distributions of continuous variables were examined for normality using the Kolmogorov-Smirnov test. When there was normality of continuous variables, the one-way ANOVA test and unpaired t-tests were used to compare differences. Otherwise, the Kruskal-Wallis one-way analysis of variance and Mann-Whitney U-test were used. Correlation between selected metabolites concentrations by ^1^H-NMR and enzymatic colorimetric methods were assessed with Pearson coefficients (or Spearman for non-normal distributions). To initially evaluate the ability of metabolites in distinguishing cases from controls, orthogonal partial least square discriminant analysis (OPLS-DA) was performed based on multivariate, supervised, discriminant analysis of spectral vectors – *Automatic Spectroscopic data Categorization by cLustering ANalysis (ASCLAN)*^105^. Finally, the Jonckheere–Terpstra test was also used to test if the concentration of metabolites followed a ordered alternative hypothesis across severity groups (controls-SGA-FGR)^113,114^. Statistical analysis was performed using STATA 14 (Stata Corp LP, 2015, College Station, Texas) and MATLAB (MathWorks Inc, US). A p-value < 0.05 was considered to be statistically significant.

## Electronic supplementary material

Supplementary information

## Data Availability

Clinical information of the patients included, spectrum data and associated protocols are promptly available to readers without undue qualifications in material transfer agreements.
