# Supplementary material for: Metabolic profiling and targeted lipidomics reveals a disturbed lipid profile in mothers and fetuses with intrauterine growth restriction
Source: Sci Rep. 2018 Sep 11;8:13614. doi: 10.1038/s41598-018-31832-5 (PMC6134091; doi:10.1038/s41598-018-31832-5)
Supplement: Supplementary file 1 — Supplementary information [file 41598_2018_31832_MOESM1_ESM.docx]

**Metabolic profiling and targeted lipidomics reveals a disturbed lipid profile in mothers and fetuses with intrauterine growth restriction**

Jezid Miranda, MD, MSc^1^, Rui V. Simões, PhD^1^, Cristina Paules, MD^1^, Daniel Cañueto^2^, Miguel A. Pardo-Cea, PhD^3^, María L. García-Martín, PhD^4^, Francesca Crovetto, MD, PhD^1^, Rocio Fuertes-Martin^2,3^, Monica Domenech, MD, PhD^5^, María D. Gómez-Roig MD, PhD^1^, Elisenda Eixarch, MD, PhD^1^, Ramon Estruch, MD, PhD^5^, Stefan R. Hansson, MD, PhD^6^, Nuria Amigó, PhD^3^, Nicolau Cañellas, PhD^2,7^, Fatima Crispi, MD, PhD^1*^, Eduard Gratacós, MD, PhD^1^

^1^Fetal i+D Fetal Medicine Research, BCNatal – Barcelona Center for Maternal-Fetal and Neonatal Medicine (Hospital Clínic and Hospital Sant Joan de Deu), Institut Clinic de Ginecologia, Obstetricia i Neonatologia, IDIBAPS, University of Barcelona, and Centre for Biomedical Research on Rare Diseases (CIBER-ER), Barcelona, Spain.

^2^Metabolomics Platform, IISPV, DEEiA, Universidad Rovira i Virgili, Tarragona, Spain.

^3^Biosfer Teslab, Reus, Spain.

^4^BIONAND, Andalusian Centre for Nanomedicine and Biotechnology, Junta de Andalucía, Universidad de Málaga, Málaga, Spain.

^5^Hospital Clínic, Institut d'Investigacions Biomèdiques August Pi i Sunyer, University of Barcelona, Barcelona; and CIBER Fisiopatología de la Obesidad y Nutrición (CIBEROBN), Instituto de Salud Carlos III, Spain

^6^Section of Obstetrics and Gynecology, Department of Clinical Sciences Lund, Lund University, Sweden; Skåne University Hospital, Malmö/Lund, Sweden.

^7^CIBERDEM, Spanish Biomedical Research Centre in Diabetes and Associated Metabolic Disorders, Madrid, Spain.

Corresponding author: Fatima Crispi, MD, PhD. fcrispi@clinic.cat

**Supplemental material**

**Methods**

*Lipoprotein profile characterization from NMR data using Liposcale*

Briefly, particle concentration and the diffusion coefficients were obtained from the measured amplitudes and attenuation of their distinct lipid methyl group signals, using the Diff ^1^H NMR spectroscopy pulse. The methyl signal was surface fitted with nine Lorentzian functions associated with each lipoprotein subtypes: large, medium and small of the main types of lipoprotein (VLDL, LDL, IDL and HDL). The area of each Lorentzian function was related to the lipid concentration of each lipoprotein subtype, and the size of each subtype was calculated from their diffusion coefficient. The particle numbers of each lipoprotein subtype were calculated by dividing the lipid volume by the particle volume of a given class. The lipid volumes were determined by using common conversion factors units.^1^ The unit scales for each variable measured were corrected according to enzymatic colorimetric methods tests run for the same sample, using correlation criteria, as described below.

*Lipoprotein analysis using enzymatic colorimetric methods*

The concentrations of cholesterol and HDL-c were determined by an enzymatic colorimetric method with Advia Chemistry (model 2400, Siemens), using the kits Cholesterol Liquiform (Advia CHOL, Siemens Diagnostics, USA) and D-HDL (ADVIA Chemistry, Siemens Diagnostics, USA), respectively. Plasma triglycerides were determined using the triglycerides method based on the Fossati three-step enzymatic reaction with a Trinder end point (ADVIA Chemistry Systems, Siemens Healthcare Diagnostics Inc.). LDL-C was calculated by the Friedewald equation: [LDL-C (mmol/L) = TC (mmol/L) − HDL-C (mmol/L) −TG (mmol/L)/2.22]^2^, using HDL-C from each manufacturer and TC and TG, as described above. The results were used to validate the quantitative analysis of the Liposcale test (**Supplemental material** - **Figure S2**).

*Phosphatidylcholines compounds and glycoprotein peak deconvolution from NMR data*

The Diff NMR data was also used to quantify choline compounds (3.3-3.18 ppm) and glycoproteins (2.15-1.9 ppm), based on peak deconvolutions. The choline region in the raw data was deconvoluted with four Lorentzian functions and two baseline Lorentzian functions, to reproduce the raw spectrum (**Fig. S3A**). To obtain the parameters related to the glycosylated proteins, the peak associated with blood glycoproteins was used, which is between 2.19 and 1.90 ppm of chemical shift in the frequency spectrum of 1H NMR.

Through a mathematical treatment of the signal, the decomposition of the envelope of the glycoprotein peak was obtained with a sum of four Lorentzian analytical functions. The three functions were classified from highest to lowest chemical shift in 1 (Glyc-B, Sialic acid); 2 (Glyc A, N-acetylglucosamine bonds) and 3 Glyc-L (lipid-associated) (**Fig. S3B**). For each of these functions the total area and height (proportional to concentration), position (characteristic of the magnetic environment) and width (related to the flexibility and the state of aggregation of the molecules that generate the signal) were determined.

**Figure S1.** Spectral profiling for lipoproteins and low molecular weight metabolites.


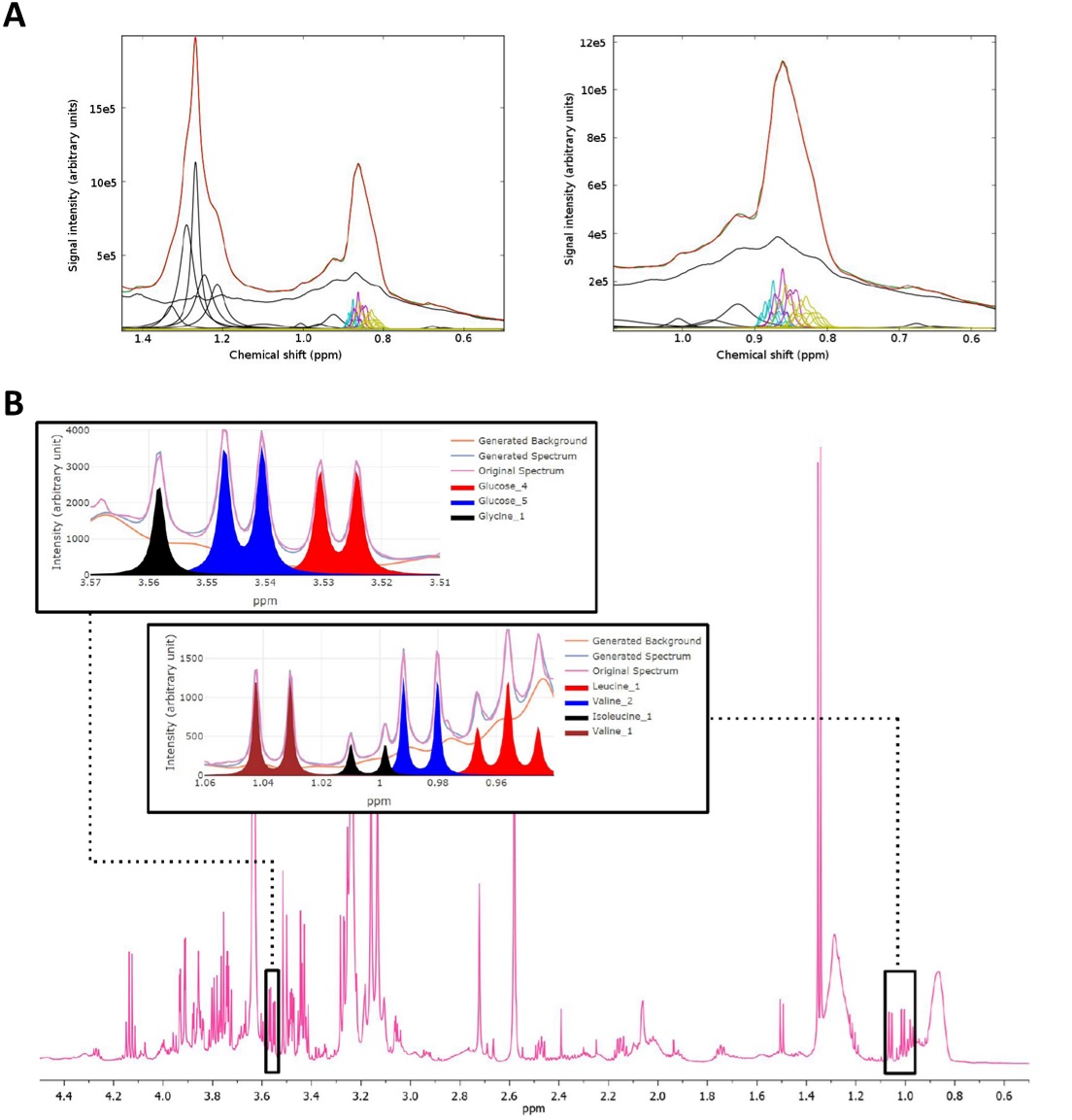


A) Liposcale analysis (lipoproteins, Diff spectra): Original spectrum (red), sum of deconvolution functions (green), base functions (black), and VLDL, LDL and HDL functions (blue, magenta and cyan, respectively). Left side: Methylene/methyl regions. Right side: methyl region. B) Dolphin analysis (LMW metabolites, CPMG spectra): original spectrum (pink), generated spectrum (blue), background (orange). The inserts on the top show enlarged spectral regions with specific deconvolutions for glycine (black) and glucose (red and blue), and for branched amino acids (leucine, red: valine, blue and brown: and isoleucine, black).

**
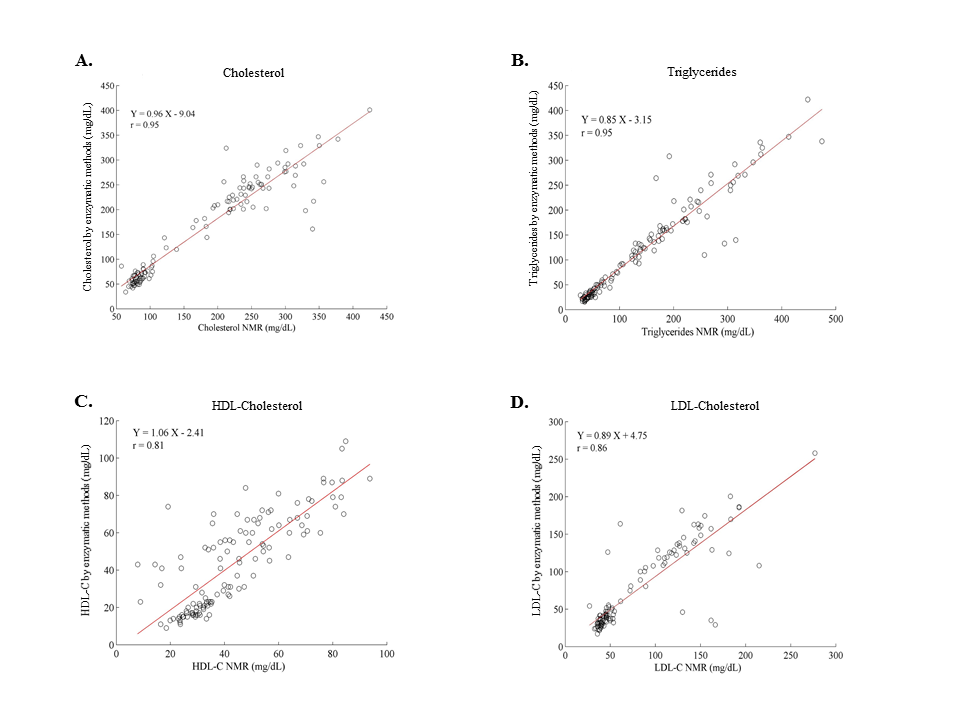
Figure S2.** Correlation of lipoprotein and triglycerides concentrations obtained with two analytical techniques: NMR-based metabolic fingerprinting and profiling and enzymatic colorimetric methods.

Commercially available kits were used in order to quantitatively analyze total cholesterol, HDL, LDL, VLDL, and free fatty acids concentrations in maternal and cord blood plasma. The quantification in each of the samples was used to calculate the Pearson’s correlation coefficient (r) between the two analytical techniques. A: total cholesterol; B. Triglycerides; C: HDL-cholesterol and D: LDL-cholesterol.

**Figure S3.** Analytical functions corresponding to the deconvolution of the choline and glycoprotein signals.

**
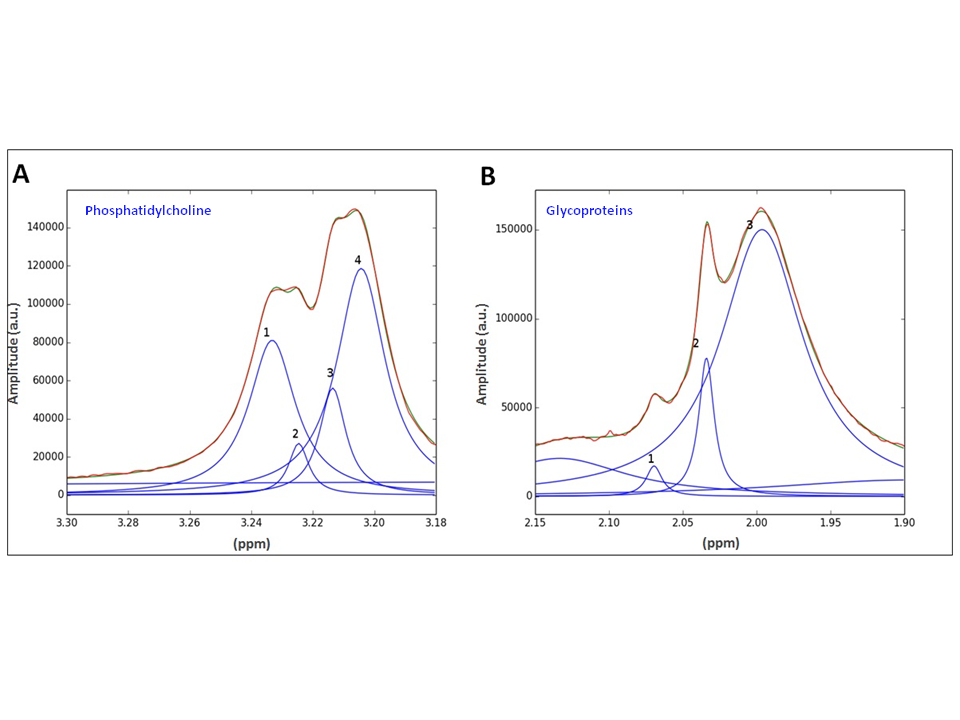
**

The choline region in the raw data was deconvoluted with four Lorentzian functions and two baseline Lorentzian functions, to reproduce the raw spectrum (Fig. S3A). For the glycoprotein region, three Lorentzian functions were used for the fitting based on the chemical shifts detected, and two baseline Lorentzian functions. The three functions were classified from highest to lowest chemical shift in 1 (Glyc-B, sialic acid); 2 (Glyc A, N-acetylglucosamine bonds) and 3 Glyc-L (lipid-associated) (Fig. S3B).

**References**

1. Jeyarajah, E. J., Cromwell, W. C. & Otvos, J. D. Lipoprotein Particle Analysis by Nuclear Magnetic Resonance Spectroscopy. *Clinics in Laboratory Medicine* **26,** 847–870 (2006).

2. Friedewald, W. T., Levy, R. I. & Fredrickson, D. S. Estimation of the concentration of low-density lipoprotein cholesterol in plasma, without use of the preparative ultracentrifuge. *Clin. Chem.* **18,** 499–502 (1972).
